# Forensic child & adolescent psychiatry and psychology in Europe

**DOI:** 10.1186/s13034-024-00756-6

**Published:** 2024-06-14

**Authors:** Cyril Boonmann, Klaus Schmeck, Andreas Witt

**Affiliations:** 1 Child and Adolescent Psychiatric Research Department (UPKKJ), University Psychiatric Hospitals, University of Basel, Basel, Switzerland; 2 Department of Forensic Child and Adolescent Psychiatry (UPKF), University Psychiatric Hospitals, University of Basel, Basel, Switzerland; 3 Department of Child and Adolescent Psychiatry – LUMC Curium, Leiden University Medical Center, Leiden, The Netherlands; 4 Department of Child and Adolescent Psychiatry, University Psychiatric Services Berne, Berne, Switzerland

*In collaboration with Ricardo Barroso (Organizing Committee EFCAP 2024 & EFCAP), Nélio Brazão (Organizing Committee EFCAP 2024), Richard Church (RCPSYCH & EFCAP), Annemiek Harder (EFCAP-NL), Riittakerttu Kaltiala (EFCAP-FI), Evi Koenekoop (LUMC Curium), Madleina Manetsch (EFCAP-CH), Kees Mos (EFCAP-NL), Eva Mulder (EFCAP-NL), Belinda Plattner (EFCAP), Daniel Rijo (Organizing Committee EFCAP 2024), Chijs van Nieuwenhuizen (EFCAP), Roberta Vacondio (EFCAP), Dirk van West (EFCAP), Marco Zanoli (EFCAP)*.

Forensic child and adolescent psychiatry is a relatively young field that has evolved as a specialized branch of child and adolescent psychiatry analogous to forensic psychiatry. Until now, in most countries, forensic child and adolescent psychiatry remains still in a niche. There are only very few journals with a focus on forensic psychiatry and psychology, and none in the field of forensic child and adolescent psychiatry. Therefore, the offer of Child and Adolescent Psychiatry and Mental Health (CAPMH) in 2011 to start a thematic series under the auspices of European Association for Forensic Child and Adolescent Psychiatry, Psychology and other involved Professions (EFCAP) was a tremendous chance to get forensic child and adolescent psychiatry out of its niche. CAPMH was founded in 2007 and was the first independent, open access, online journal in the field [31]. The journal is established in the field and currently holds a 2 year Impact Factor of 5.6 and a 5 year Impact Factor of 5.0 (2022 Citation Impact).

Traditionally, the primary function of forensic child and adolescent psychiatry has been, and continues to be, the forensic evaluation and treatment of children, and especially adolescents, who have been committed to mental health services and/or institutions on the basis of civil or criminal law. Therefore, forensic child and adolescent psychiatry is characterized by a high inter-institutional cooperation, especially with the legal system that entail a variety of challenges and opportunities.

The relative youth of forensic child and adolescent psychiatry is reflected in the fact that it was not until 1997 that the umbrella organization EFCAP was founded, followed by the establishment of active national groups in the Netherlands (EFCAP-NL; for more information: see Box 1), Finland (EFCAP-FI; for more information: see Box 2) and Switzerland (EFCAP-CH; for more information: see Box 3). EFCAP also cooperates closely with the Adolescent Forensic Psychiatry Special Interest Group of the Royal College of Psychiatrists (RCPSYCH) in the United Kingdom (For more information: see Box 4), and adolescent forensic psychiatrists and psychologists from various countries such as Austria, Belgium, Cyprus, Czech Republic, France, Germany, Hungary, Italy, Lithuania, Portugal and Spain support EFCAP and its goals.

## Box 1: EFCAP NL (Kees Mos, Eva Mulder & Annemiek Harder)


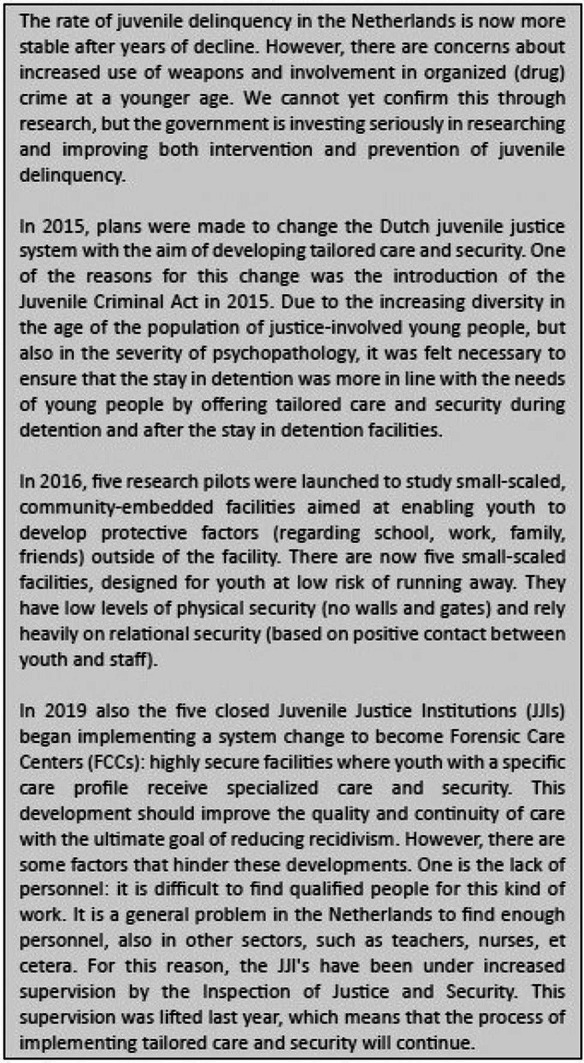


## **Box 2: EFCAP FI (Riittakerttu Kaltiala)**

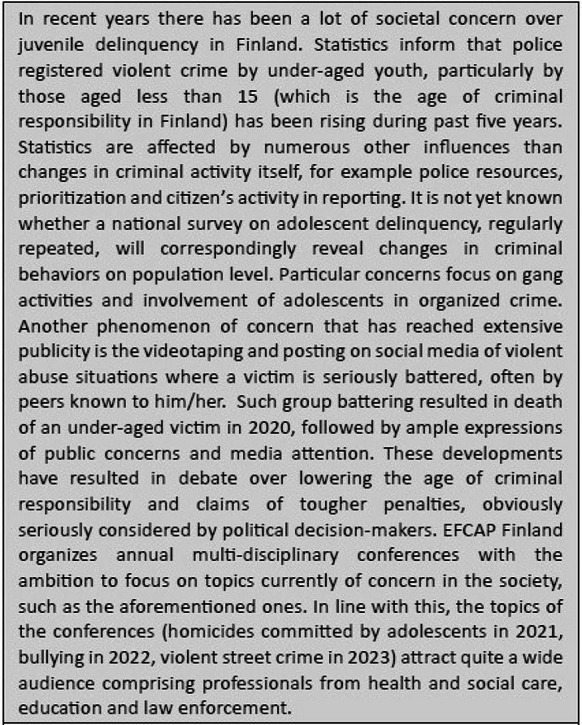



## **Box 3: EFCAP CH (Madleina Manetsch)**

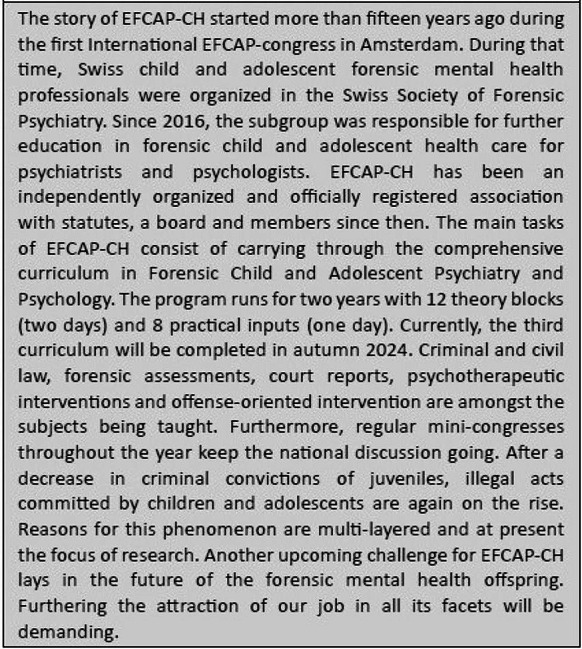



## **Box 4: RCPSYCH (Richard Church)**


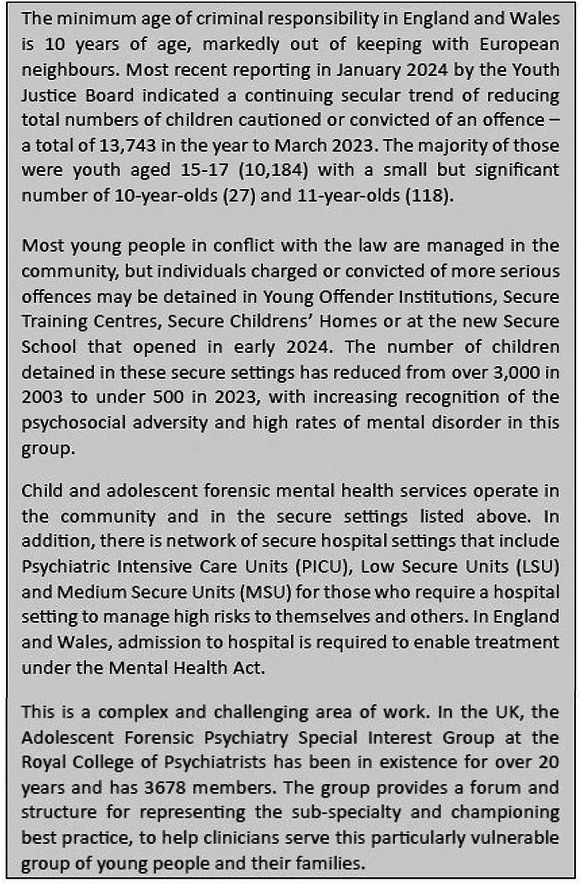


One goal of EFCAP is to bring together experts and various stakeholders (e.g., experts by experience, practitioners, scientists, policy makers) in the field of forensic child and adolescent psychiatry and psychology to learn from one another. In doing so, the scientific and political goal is a better understanding of young people in forensic settings so that services to these young people and their families can better meet their needs, protect their development, and prevent future offending behavior. To facilitate this exchange, EFCAP organizes a scientific congress every two years. After congresses in Amsterdam (2008), Basel (2010), Berlin (2012), Manchester (2014), Porto (2016), Venice (2018) and Eindhoven (2022), this year’s congress will be held in the Azores (See Box 5).

## Box 5: 8th EFCAP Congress 2024 (Daniel Rijo, Ricardo Barroso & Nélio Brazão)

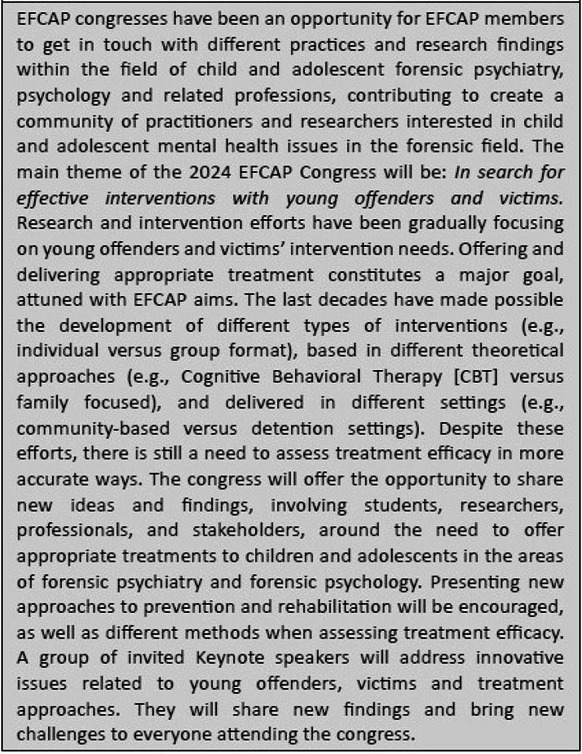



Besides the organization of congresses, the dissemination of forensic knowledge in scientific publications is a second foothold of EFCAP to foster the development of the field. The collaboration between EFCAP and CAPMH started first in the form of a thematic series [4, 9, 20, 21], and since the end of 2019 in the form of an ongoing series. Since then, 24 papers (excluding the editorials mentioned above) have been published [1–3, 5, 7, 8, 11–19, 22–30].

One publication in particular we would like to highlight is the commentary: *Overview of European forensic youth care: towards an integrative mission for prevention and intervention strategies for juvenile offenders by* Souverein and colleagues [24], that summarized the results of a panel held at the 6th EFCAP conference in Venice in 2018. This commentary concludes with an integrative mission statement for Europe:

1. Consider forensic care for juveniles in a broader socio-political perspective.
2. Invest sufficient financial resources (and demonstrate that the investment pays off).
3. Collaborate nationally and internationally.
4. Prevention is critical.
5. Involve expert youth and their parents/caregivers.

**Fig. 1:**
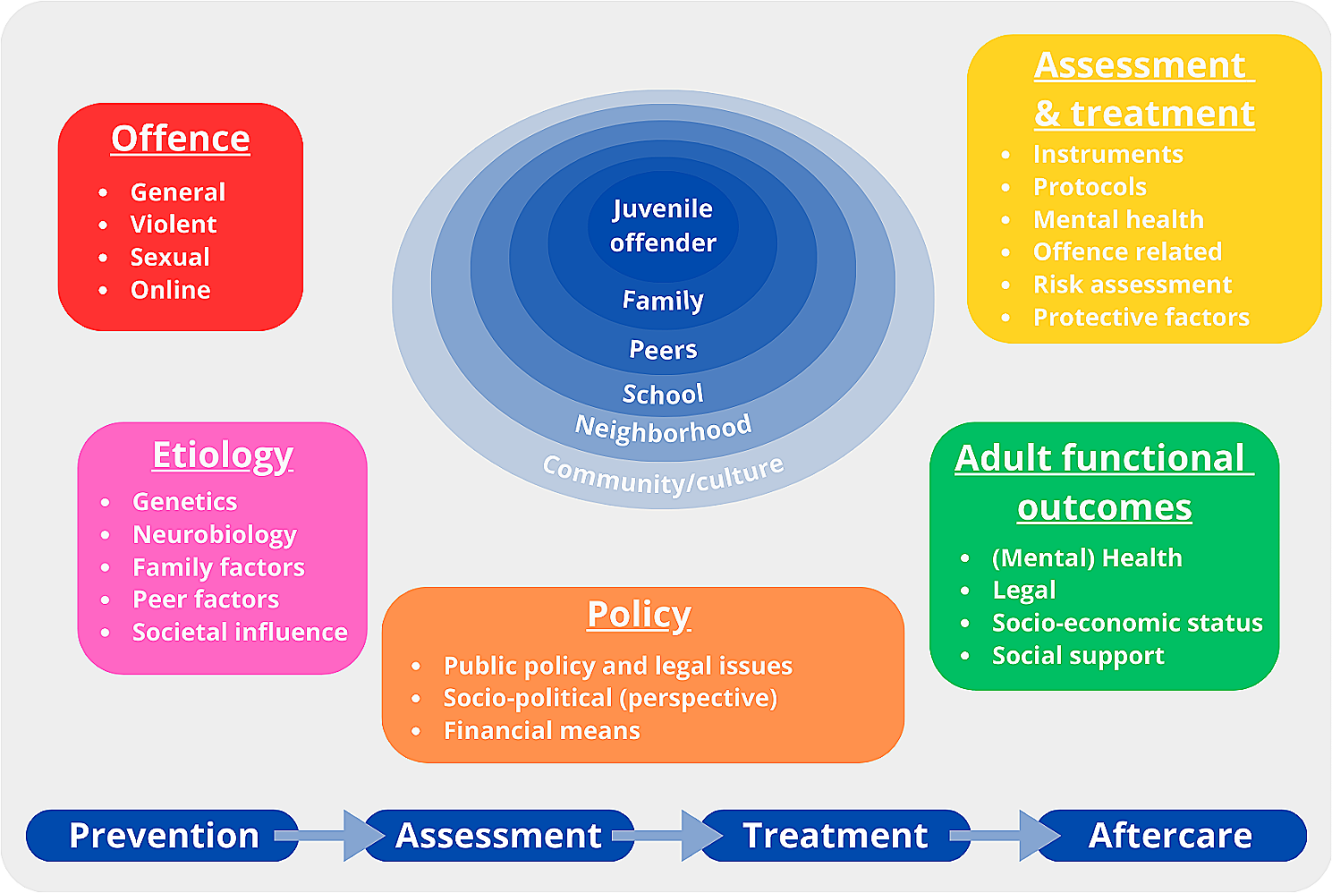
Visual summary of important topics in the field of forensic child and adolescent psychiatry

Based on the goals of EFCAP, the themes of the upcoming EFCAP Congress, and the integrative mission statement of Souverein et al. [24], Bronfenbrenner’s (1979) ecological model and Engel’s (1977) biopsychosocial model, the authors attempted to visually summarize the major themes of the field that could be of interest for publication in our ongoing series (see Fig. 1).

Looking to the future, we hope to continue our biennial conferences. In addition, we hope to welcome new national groups (or new sister organizations for countries where a forensic child and adolescent psychiatric/psychological nexus already exist). Finally, forensic child and adolescent psychiatry is an evolving field. CAPMH provides a platform for scientific communication and welcomes submissions of high quality research to further advance science and practice in the field, to better understand the needs of our patients, their families and the stakeholders and to provide better interventions.

## Data Availability

Not applicable.
