# Correction: Forensic child & adolescent psychiatry and psychology in Europe

**DOI:** 10.1186/s13034-024-00821-0

**Published:** 2024-11-25

**Authors:** Cyril Boonmann, Klaus Schmeck, Andreas Witt

**Affiliations:** 1 Child and Adolescent Psychiatric Research Department (UPKKJ), University Psychiatric Hospitals, University of Basel, Basel, Switzerland; 2 Department of Forensic Child and Adolescent Psychiatry (UPKF), University Psychiatric Hospitals, University of Basel, Basel, Switzerland; 3 Department of Child and Adolescent Psychiatry– LUMC Curium, Leiden University Medical Center, Leiden, The Netherlands; 4 Department of Child and Adolescent Psychiatry, University Psychiatric Services Berne, Berne, Switzerland

**Correction: Child and Adolescent Psychiatry and Mental Health (2024) 18:70**

10.1186/s13034-024-00756-6

Following publication of the editorial [1], the author identified the textual errors. There are five text boxes (Boxes 1–5). The text contained in the boxes have been duplicated directly below the box. This has been corrected with this erratum.

## Box 1: EFCAP NL (Annemiek Harder, Kees Mos & Eva Mulder)


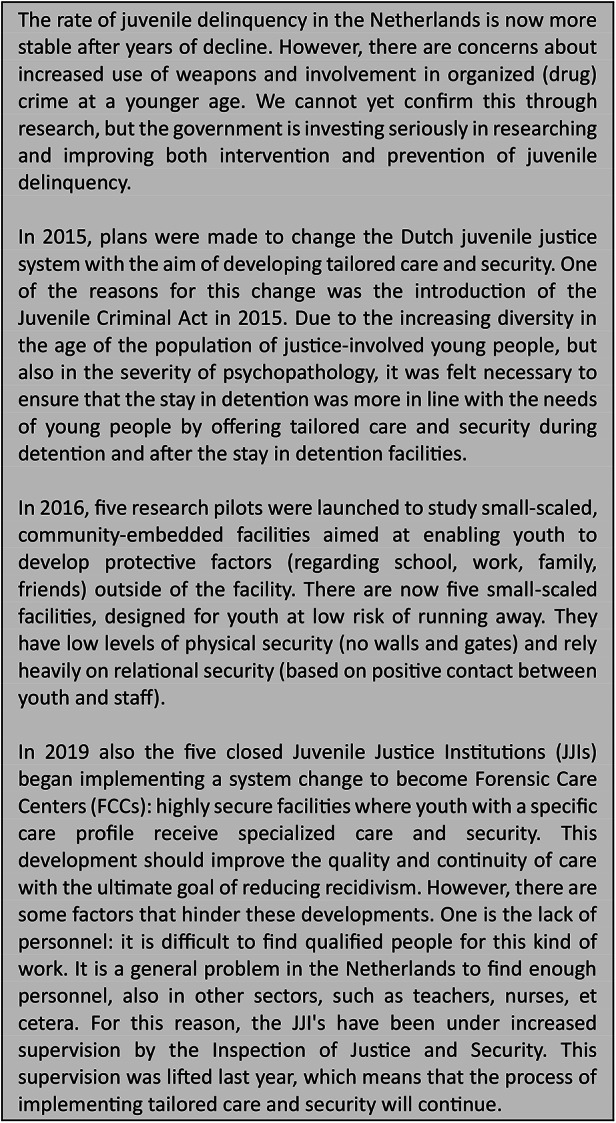


## Box 2: EFCAP FI (Riittakerttu Kaltiala)


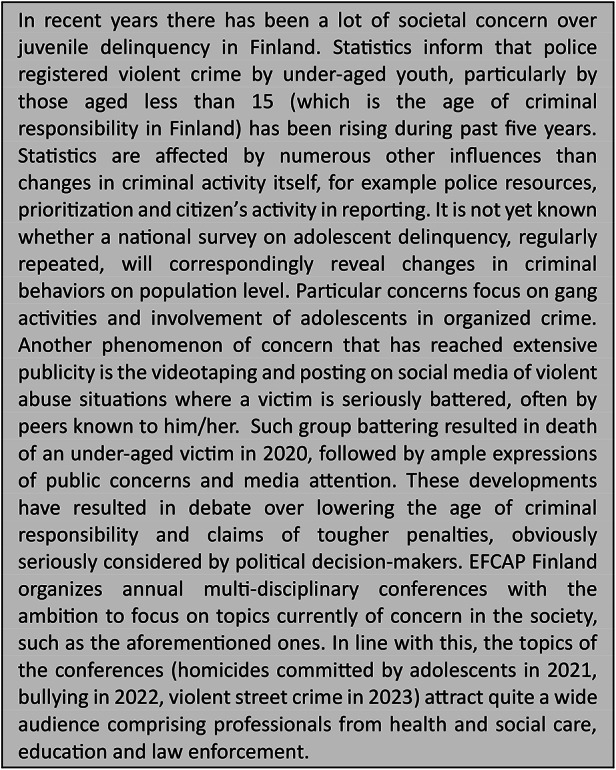


## Box 3: EFCAP CH (Madleina Manetsch)


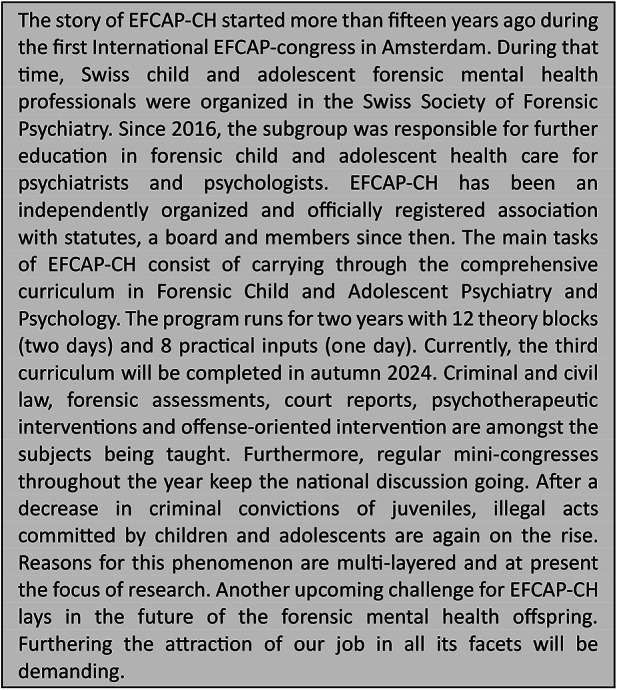


## Box 4: RCPSYCH (Richard Church)


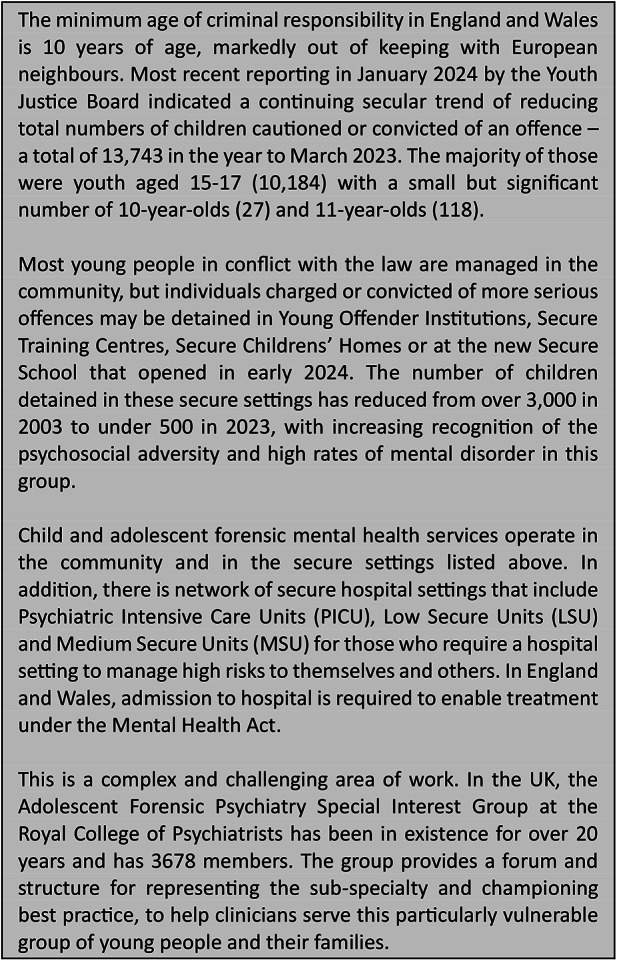


## Box 5: 8th EFCAP Congress 2024 (Daniel Rijo, Ricardo Barroso & Nélio Brazão)


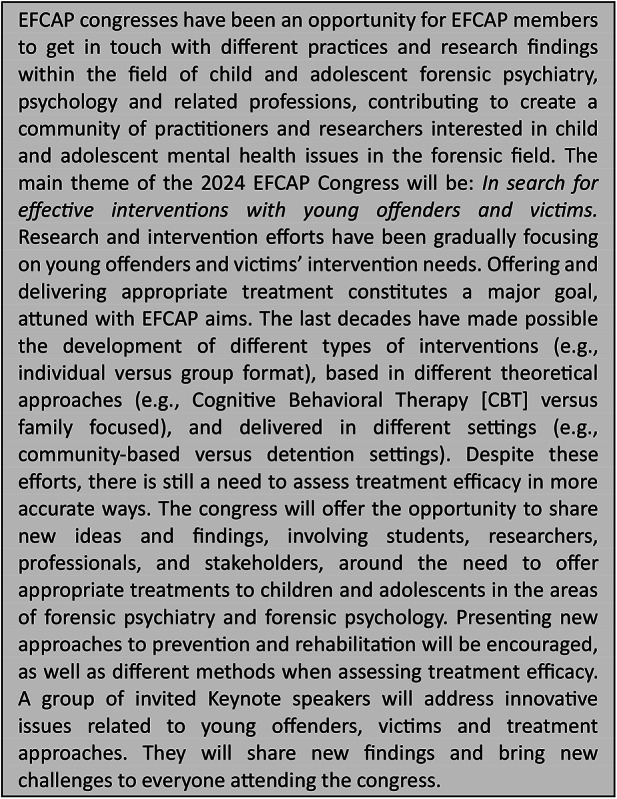


The original article has been corrected.
